# Alzheimer’s Disease as Type 3 Diabetes: Common Pathophysiological Mechanisms between Alzheimer’s Disease and Type 2 Diabetes

**DOI:** 10.3390/ijms23052687

**Published:** 2022-02-28

**Authors:** Michalis Michailidis, Despina Moraitou, Despina A. Tata, Kallirhoe Kalinderi, Theodora Papamitsou, Vasileios Papaliagkas

**Affiliations:** 1 Laboratory of Psychology, School of Psychology, Aristotle University of Thessaloniki, 54124 Thessaloniki, Greece; elfmike@hotmail.com (M.M.); despinamorait@gmail.com (D.M.); dtata@psy.auth.gr (D.A.T.); 2 Laboratory of Medical Biology-Genetics, School of Medicine, Faculty of Health Sciences, Aristotle University of Thessaloniki, 54124 Thessaloniki, Greece; roey111@hotmail.com; 3 Histology and Embryology Department, Faculty of Medicine, Aristotle University of Thessaloniki, 54124 Thessaloniki, Greece; thpapami@auth.gr; 4 Department of Biomedical Sciences, School of Health Sciences, International Hellenic University, 57400 Thessaloniki, Greece

**Keywords:** amyloid beta, Alzheimer type 3 diabetes, inflammation and cognition, brain insulin resistance, type 2 diabetes mellitus

## Abstract

Globally, the incidence of type 2 diabetes mellitus (T2DM) and Alzheimer’s disease (AD) epidemics is increasing rapidly and has huge financial and emotional costs. The purpose of the current review article is to discuss the shared pathophysiological connections between AD and T2DM. Research findings are presented to underline the vital role that insulin plays in the brain’s neurotransmitters, homeostasis of energy, as well as memory capacity. The findings of this review indicate the existence of a mechanistic interplay between AD pathogenesis with T2DM and, especially, disrupted insulin signaling. AD and T2DM are interlinked with insulin resistance, neuroinflammation, oxidative stress, advanced glycosylation end products (AGEs), mitochondrial dysfunction and metabolic syndrome. Beta-amyloid, tau protein and amylin can accumulate in T2DM and AD brains. Given that the T2DM patients are not routinely evaluated in terms of their cognitive status, they are rarely treated for cognitive impairment. Similarly, AD patients are not routinely evaluated for high levels of insulin or for T2DM. Studies suggesting AD as a metabolic disease caused by insulin resistance in the brain also offer strong support for the hypothesis that AD is a type 3 diabetes.

## 1. Introduction

Alzheimer’s disease (AD) is a chronic neurodegenerative disease that is the most common type of dementia and is characterized by impaired memory and cognitive ability, as well as changes in behavior and personality [1]. According to recent reports, 5.8 million Americans aged 65 and older currently suffer from AD, a number which is projected to rise to 13.8 million by mid-century in the US alone [2].

Brain lesions that occur in AD are accompanied by synaptic dysfunction, neurodegeneration and neuronal disorders, while the disease is characterized by extracellular plaques of insoluble β-amyloid protein and intracellular neurofibrillary tangles (NFTs) of hyperphosphorylated tau protein [3]. The exact cause of AD is not fully understood; there is therefore currently no effective treatment despite the large number of clinical studies that have been performed [4]. The strongest genetic risk factor for AD is the e4 allele of apolipoprotein E (APOE4) relative to the e3 allele and the protective e2 allele [5,6]. APOE is a lipid-binding protein with a key role in cholesterol homeostasis [7].

It is estimated that the global diabetes mellitus (DM) prevalence will rise from 9.3% (463 million people) in 2019 to 10.2% (578 million) by 2030 and 10.9% (700 million) by 2045 [8]. Type 2 diabetes mellitus (T2DM) is always characterized by abnormal blood glucose levels, a phenomenon caused by the general dysfunction between the action and secretion of insulin. This dysfunction means that the secretion of insulin by the β-cells of the pancreatic islets (β-cell dysfunction) as well as the action of insulin on insulin-sensitive tissues such as the liver, adipose tissue and muscle do not function normally for the body (insulin resistance) [9]. Insulin resistance affects the way insulin works in target tissues and cells, leading to the well-known phenomenon of hyperglycemia, a condition in which too much glucose circulates in the blood plasma and characterizes T2DM [9] T2DM has also been associated with an increased risk of dementia [10,11] and of AD by 45–90% [12,13] as well as an increased risk of AD in patients with T2DM [14].

In recent years, with the accumulation of findings suggesting that AD may represent a brain-specific form of DM (type 3 diabetes) [15,16,17], there has been a growing interest in the role of β-amyloid and tau protein in the peripheral nervous system and its organs, as well as in inducing insulin resistance [18,19]. Studies have shown that AD and T2DM share many common pathophysiological mechanisms associated with insulin resistance, such as oxidative stress, insulin signaling disorder, mitochondrial dysfunction, neuroinflammation, advanced glycosylation end products (AGEs) and metabolic syndrome.

The purpose of this review is to discuss the common molecular and cellular mechanisms between AD and T2DM, as well as the case of AD as type 3 diabetes.

## 2. Materials and Methods

### 2.1. Search of the Literature

**Figure 1:**
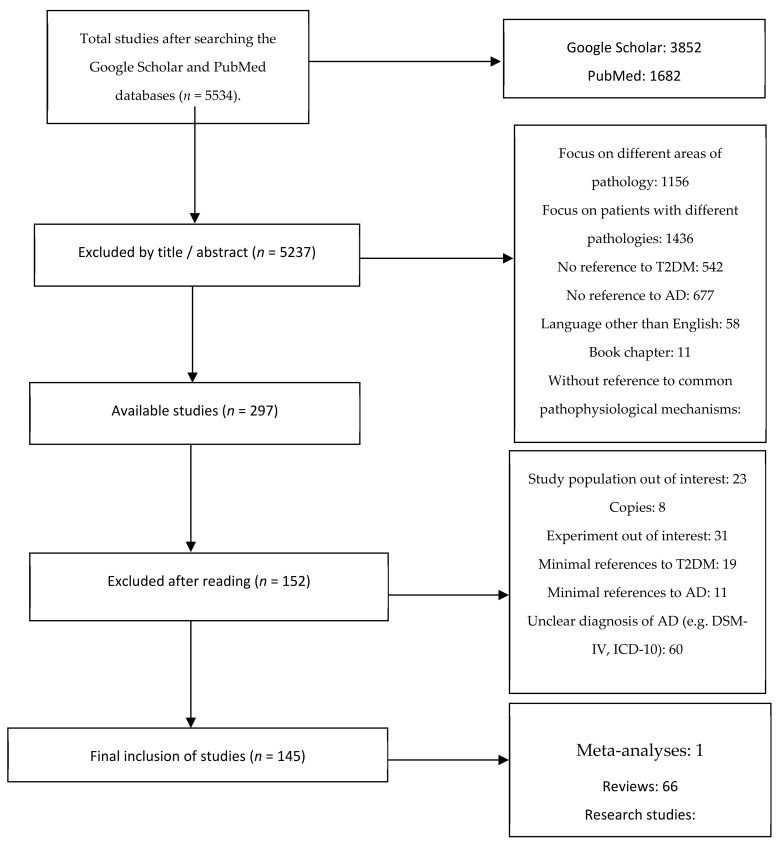
PRISMA flowchart of study selection.

A detailed literature search was conducted in the databases PubMed and Google Scholar using the following terms: “Amyloid beta”, “Alzheimer Type-3-Diabetes”, “Inflammation and cognition”, “Brain insulin resistance”, and “Type 2 diabetes mellitus”. Studies published from 2000 to 2020 were included following the Preferred Reporting Items for Systematic Reviews and Meta-Analyses (PRISMA) flow diagram (Figure 1). Two of the authors independently screened all titles and abstracts. The remaining articles were assessed for eligibility based on the full text.

### 2.2. Inclusion and Exclusion Criteria

Emphasis was placed on randomized controlled trials, while systematic reviews and meta-analyses related to the subject were also included. Selected randomized controlled trials have usually had cognitive function improvement or decline, or AD or AD progression, as an outcome. Experimental studies were also included if they provided important evidence of the basic mechanisms by which T2DM and AD might be linked.

We included studies that used internationally recognized criteria for AD diagnosis (i.e., Diagnostic and Statistical Manual of Mental Disorders (DSM) or National Institute of Neurological and Communicative Disorders and Stroke and the Alzheimer’s Disease and Related Disorders Association criteria for Alzheimer’s disease (NINCDS-ADRDA)). Research focusing on different areas of disease pathology (e.g., T2DM and depression), as well as research focusing on a population out of the review’s interest (e.g., patients receiving specific medication) were excluded. Only articles written in English were included.

## 3. Results

### 3.1. Common Pathophysiological Mechanisms between AD and T2DM

Several studies have reported common links between AD and T2DM (A sample is shown in Table 1).

### 3.2. Role and Production of Insulin in the Brain

The increase in insulin concentration in the cerebrospinal fluid (CSF) was first experimentally confirmed by Margolis and Altszuler [28] in mice. The highest percentage of insulin in the brain is derived from pancreatic peripheral neural insulin [29]. The way insulin enters the brain is mainly through a selective transport along the capillary endothelial cells of the blood-brain barrier (BBB) [30], which appears to be influenced by risk factors such as DM, obesity, inflammation and blood triglyceride levels [31].

Insulin performs many important functions in the brain related to the regulation of food intake, body weight, eating habits and homeostasis of energy [30,31]. It also appears to affect neurotransmitters and, in particular, the density of their receptors, while also acting as a regulator of long-term memory enhancement (LTP) and long-term memory suppression (LTD) [31].

Previously, the main source of insulin in the brain was believed to be of peripheral origin, although today various hypotheses are made as to whether the brain produces its own insulin [32,33,34]. Insulin mRNA expression has been found in specific areas of the brain in mice, and insulin peptide production has also been observed in cultured neurons but not in glial cells from mice brains [35,36,37]. In contrast to studies that detected insulin mRNA expression, others did not find corresponding mRNA expression in significant amounts to confirm insulin production in the brain [38,39,40,41].

### 3.3. Insulin Resistance and β-Amyloid

AD and T2DM are both diseases that involve the formation of amyloid in various parts of the body [42,43]. Beta-amyloid molecules can, through their accumulation, form various forms of flexible soluble oligomers. Oligomers are toxic to nerve cells, while misfolded oligomers can transmit the wrong folding to their neighbors acting as prions [44,45,46,47]. The aggregation of the amyloid peptide into oligomers or fibrils, in turn, is involved in the progression of AD [48].

Reduced levels of IGF-1(insulin-like growth factor) [49] and insulin resistance may result in reduced IGF-1 and insulin brain uptake, causing β-amyloid accumulation. Consequently, increased β-amyloid levels antagonize insulin and IGF-1 receptor binding, which results in the secretion of inflammatory agents and the onset of insulin resistance [50]. Moreover, these factors of insulin dysfunction and inflammation, in combination with oxidative stress, enhance the toxicity and concentration of β-amyloid [51] in a pathological feedback cycle. Hence, the dysregulation of insulin signaling can affect the metabolism and function of APP, eventually leading to the accumulation of Aβ in the cell, which is a major cause for neurodegeneration in AD [52]. Evidence shows that APP is essential for maintaining a healthy glycemic regulation, and it has been observed that APP knockdown mice develop metabolic disorders [53].

Insulin stimulation plays a key role in the clearance of β-amyloid by preventing extracellular accumulation and the final formation of fibrils and plaques [54]. Insulin-degrading enzyme (IDE) is the major degradation peptidase of β-amyloid, and due to its ability to degrade insulin, amylin and β-amyloid, it is thought to play a linking role between insulin resistance and AD [55]. Previous studies have underlined the vital role of the IDE. For example, Qiu and colleagues located the IDE in extracellular domains via antibodies and verified that in insulin resistance states where insulin levels are high, the IDE is diverted to insulin degradation, thereby reducing β-amyloid degradation and its toxic accumulation [56]. In mice, it has been shown that increased γ-secretase activity due to insulin resistance leads to increased amyloid in the brain, as well as decreased activity of the IDE [57]. In addition, a decrease in β-amyloid clearance was observed in high-dose insulin mice [58], whereas in mice with absence of both alleles of the insulin degradation enzyme gene, a significant reduction of up to 50% was found in the degradation of β-amyloid, resulting in its abnormal concentration in the brain [59]. In patients with AD who carry the APOE4 gene, the expression of IDE is quite reduced in areas of the brain such as the hippocampus [60]. The expression and activity of this enzyme appears to be reduced in studies with patients who have been diagnosed with genetically inherited forms of AD [61,62].

### 3.4. Insulin Resistance and Tau Protein

In its normal state, tau protein is responsible for microtubule stability and regulating intracellular signaling [63,64,65,66].

The tau protein hypothesis argues that tau protein hyperphosphorylation results in the transformation of normal tau protein into paired helical fibers (PHF) and neurofibrillary tangles (NFTs) leading to the pathophysiology of AD [67].

Tau protein plays an important role in the peripheral nervous system [19,42,68] as well as in inducing insulin resistance and T2DM [42]. It has been shown that the pathology of neurofibrillary tangles can occur in the pancreas [19], while the presence of tau protein and β-amyloid in the pancreas has also been proven after analysis of pancreatic tissue from 21 autopsy cases of patients with T2DM [19]. However, in another relevant study [21], there was no increase in β-amyloid in the brain of patients with T2DM, but when β-amyloid was present it increased according to the severity of DM. Additionally, the presence of tau protein hyperphosphorylation has also been demonstrated in the pancreatic islets of transgenic mice with T2DM and AD [69].

The rate of tau protein aggregation relies heavily on tau protein phosphorylation and, consequently, on the function of certain kinases and phosphatases. Insulin and IGF signaling impairment may result in decreased tau protein expression and hyperphosphorylation due to overactivity by certain kinases and phosphatases [70,71,72], with GSK-3β (glycogen synthase kinase 3) being the most important. GSK-3β has been shown to lead to tau protein phosphorylation; it has also been shown that insulin secretion and action reduce GSK-3β activity by activating the PI3 kinase signaling pathway [73]. According to human and animal studies, the phosphatase PP2A is involved in both AD and T2DM, and its expression is suppressed by insulin administration [74,75].

### 3.5. Neuroinflammation

Neuroinflammation occurs in the early stages of AD [76] while its manifestation includes the increase of inflammatory cytokines and the penetration of microglial cells in the area of amyloid plaques [77,78]. It has been shown that neuroinflammation contributes to the pathology of AD through oxidative damage [79], tau protein hyperphosphorylation [80], β-amyloid accumulation [78] and by causing cholinergic system dysfunction [81]. The presence of high levels of inflammatory cytokines, such as interleukin-1β (IL-1β), IL-6 and interferon-gamma in close proximity to β-amyloid plaques and macrophage cells supports the important role of neuroinflammation in the pathology of AD [82,83].

Neuroinflammation is considered by a large number of studies as one of the main causes of insulin and IGF-1 resistance observed in the brain of AD patients [49,84]. Peripheral insulin resistance induced by T2DM and obesity results in the production of high levels of cytotoxic lipids, which in turn cross the blood-brain barrier and cause neuroinflammation and insulin resistance in the brain [85]. One of the most important processes involved in neuroinflammation is the beta-amyloid-induced activation of microglial cells [86], which in turn leads to the release of various inflammatory cytokines such as interleukin-6 and tumor necrosis factor alpha (TNF-a). The important thing with these inflammatory processes is that they mediate the relationship between T2DM and AD [85], while they have also been shown to cause a decrease in synaptic function, inhibition of hippocampal neurogenesis and neuronal death, which enhances the theory that T2DM may act as a precursor to the activation of inflammatory substances that lead to neuroinflammation and ultimately to AD [86]. Research on chronic stress suggests that interleukin-6 is involved in the progression of AD as well as in the pathology of T2DM [87].

It has been argued that reduced cerebral glucose metabolism begins in the early stages of AD [88] and that glial cells detect this glucose deficiency [89]. This deficiency, supported by systemic hyperinsulinemia, leads to the perpetuation of inflammation through its inhibition of AMPK (5’ AMP-activated protein kinase) [89]. Due to this inflammatory process and the presence of increased levels of inflammatory cytokines, the pathology of AD occurs [78].

Although neuroinflammation is an integral part of AD pathophysiology [49,76,77,85], its role in inducing brain insulin resistance and in mediating pathology in T2DM and AD have not been fully established. There is currently no scientific evidence that anti-inflammatory therapies work against AD.

### 3.6. Oxidative Stress and Mitochondrial Dysfunction

There are indications that insulin resistance in T2DM may be considered as a factor that increases and promotes oxidative stress leading to the neurodegeneration of AD [90].

Oxidative damage results from an imbalance in favor of the highly reactive forms of oxygen and nitrogen, which constitute the reactive forms of oxygen. It leads to oxidation of proteins, lipids and DNA, causing damage to the mitochondria, as well as induction of genetic mutations in DNA/RNA [91]). Reactive forms of oxygen enhance cell apoptosis and promote the production of inflammatory cytokines [91,92].

Mitochondrial dysfunction and the overproduction of reactive oxygen species play a vital role in the development of T2DM pathology [93]. Under conditions of insulin resistance, increased derivatives of oxidative reactions are observed in blood plasma [23,94,95], in adipose and brain tissue, muscle and liver [22,23,96,97]. It has also been shown that T2DM deregulates mitochondrial function in mice, as well as impairs neurite outgrowth through JAK/STAT3 modulation of mitochondrial bioenergetics [27].

The human brain is particularly vulnerable to oxidative stress damage [98,99] because although it constitutes a small fraction (less than 2%) of total body weight, it consumes more than 20% of oxygen of the organism. Low levels of antioxidants, the presence of high levels of oxidizing metal ions (Fe^2+^, Cu^2+^), as well as the ease of hyper-oxidation of polyunsaturated fatty acids (PUFAs) of the brain cells’ membrane are additional factors that make the brain vulnerable to oxidative-stress-induced damage [23,98,99].

Active forms of oxygen produced by oxidative stress and the resulting damage are most pronounced in AD in the form of attenuation of ATP (adenosine triphosphate) production, neuronal degeneration and the presence of oxidative stress products in neurofibrillary tangles [79]. It has been shown that β-amyloid, which functions as the major component of amyloid plaques between nerve cells in AD with high concentrations in mitochondria, can produce reactive forms of oxygen in the presence of metal ions such as Fe^2+^ and Cu^2+^ [100].

Thus, while some have argued that β-amyloid precedes oxidative stress [100], many studies suggest that oxidative stress may be a precursor to β-amyloid and tau protein concentration [101,102]. It has been proposed that oxidative stress and insulin resistance can lead independently to the accumulation of β-amyloid and tau protein [103,104], thus implying a strong correlation with AD pathology.

From all the above-mentioned studies there seems to be a network of integral links between insulin resistance, AD and oxidative stress, which consists in a positive feedback system with a rather complex grid of causes and relationships.

### 3.7. Advanced Glycosylation End Products (AGEs)

Advanced glycosylation end products (AGEs) play a significant role in the development of AD. These products are protein molecules or peptides that are formed as a result of the Maillard reaction, meaning they become glycated as a result of their exposure to sugars [105]. AGEs accumulate in various cells as products of the normal course of aging, but their rate of accumulation is increased in patients with DM [106,107]. AGEs interact with their receptors (RAGE) and activate several signaling mechanisms that cause increased oxidative stress and organ damage, leading to complications [108].

AGEs promote the aggregation of amyloid oligomers, thus contributing to the neurotoxic effects observed in AD [109]. Furthermore, it also appears that tau protein glycation may promote the formation of paired helical fibers [110]. What has also been suggested is the coexistence of AGEs and APOE4 gene in the amyloid plaques of patients with dementia and specifically AD [111]. These products are clearly neurotoxic molecules, since as Sato et al. (2006) showed, they reduced cell viability when added to cortical neurons [108]. In addition, it has been shown that AGEs can produce free radicals by initiating NADPH oxidase (NOX) activity, which is the major source of reactive oxygen species in glial cells and neurons [112].

In conclusion, AGEs are a very important link between insulin resistance and neurodegeneration, especially in cases of hyperglycemia [23,112,113].

### 3.8. Metabolic Syndrome

Research over the past decade has linked all the key features of metabolic syndrome, such as visceral obesity [114], various metabolic problems [115,116] and insulin resistance [117] with cognitive impairment and cerebral atrophy [25,118]. In fact, insulin resistance has been highly correlated with the reduced rate of glucose metabolism in specific areas of the brain such as the frontal, temporal and parietal cortex areas in patients with T2DM [119,120]. In addition, in the white and gray matter of patients with T2DM who also suffer from mild cognitive impairment (MCI), a total cerebral decrease in FDG (18-fluoro-deoxyglucose) intake has been observed [121]. It has also been shown that older patients with T2DM and a history of severe hypoglycemic episodes followed up for 27 years showed a greater risk of dementia [24].

There is evidence that brain metabolism is a key feature of AD [122,123], as well as that the brain’s ability to use glucose to function properly in patients with AD is reduced by up to 25%, mainly in parts of the brain such as the hippocampus [122]. This reduced metabolism of cerebral glucose in patients with AD has been repeatedly confirmed by several research studies [88,123,124]. Autopsy studies comparing patients with AD and the control group showed reduced levels of glucose transporters (GLUT) in the hippocampus, frontal cortex, parietal lobe and temporal lobe, in patients with AD [125,126]. It should be noted that in their recent research, An et al. [26] demonstrated that high levels of glucose in brain tissues, as well as low levels of glucose-3 transporters (GLUT3), are associated with AD pathology. These metabolic disorders, which accompany AD even from its early stages [26,127], lead to exacerbations of insulin signaling disorders, glucose use and insulin resistance problems [20,34,128], such as decreased acetylcholine (ACh) levels [20], with further aggravation of the pathological condition for those carrying the genetic risk factor APOE4.

### 3.9. Amylin

Islet amyloid polypeptide (IAPP), otherwise known as amylin, is a hormone that is secreted along with insulin by the β-cells of the pancreatic islets [129] and helps induce satiety in order to regulate blood sugar, inhibit glucagon secretion and delay gastric emptying [130].

Like insulin, amylin levels are higher than normal in patients with obesity or insulin resistance, while the coexistence of hyperinsulinemia and hyperamylinemia is common [131,132]. Amylin receptors [132,133,134], as well as amylin deposition [134,135] in the brains of AD patients are observed using immunofluorescence. Amyloid plaques have amylin deposits and mixed amylin-β-amyloid plaques [134,135,136].

Some studies indicate a significant involvement of amylin in the additional amyloid formation in the central nervous system, especially under conditions of hyperamylinemia [137]. Amylin is incorporated into neurons and acts on them with the same toxicity that is observed in the pancreatic islets in individuals with T2DM, causing intracellular oxidative stress and inflammatory responses [136].

Therefore, the understanding of pathological feedback between AD and T2DM is enhanced by elucidating the molecular relationships between amylin and β-amyloid, as it appears that amylin found in the brains of patients with AD [134,136] may cause amyloid formation [134,135,136,137,138,139,140,141,142] through heterologous interaction. Amylin resistance and hyperamylinemia may be targets of the therapeutic approach against AD [143].

## 4. Discussion

In the present review paper we attempted to collect all existing evidence on the case of AD as type 3 diabetes and the common pathophysiological mechanisms between AD and T2DM. Diabetic patients have higher incidence of cognitive impairment and dementia in particular vascular dementia and AD. The severity of cognitive impairment depends on type of diabetes, age of onset, and other comorbidities. Based on existing evidence, we now know that insulin resistance, hypoglycemia [24] and hyperglycemia are associated with cognitive decline [144] and are key features of T2DM. Insulin and IGF-1 play an important role in cognitive ability, neuronal function and development [145], as well as an important role in AD [146].

Insulin resistance in the brain as a feature of AD was first proposed in an earlier study by Hoyer and Nitsch (1989) to explain the metabolic disorders of the brain and more specifically as the main explanation for the low glucose metabolism observed during the AD [147]. Today’s evidence shows the clear role of insulin in the brain, while some studies also support the possible production of insulin in the brain. For this type of insulin resistance-induced AD, the term “type 3 diabetes” has been proposed.

As shown in this review, AD shares many common pathophysiological characteristics and signaling pathways with T2DM, such as neuroinflammation, oxidative stress, advanced glycosylation end products, mitochondrial dysfunction and metabolic syndrome. A significant involvement of β-amyloid, tau protein and amylin in both diseases is also indicated. Β-Amyloid is involved in brain insulin resistance, thus contributing to the pathological vicious cycle that forms between oxidative stress and neuroinflammation

As far as patients with T2DM are concerned, they are not routinely evaluated for cognitive outcomes and they are rarely treated for cognitive impairment. Similarly, AD patients are not routinely evaluated for high levels of insulin or for T2DM. The apparent involvement of T2DM in the pathology of AD requires a new and holistic therapeutic approach based on current data regarding this devastating disease.

In conclusion, it seems reasonable to consider T2DM family history as a possible risk factor for AD, as well as low brain glucose metabolism as an important biomarker for the development of dementia and AD. In addition, existing evidence underlines the importance of conducting frequent tests of fasting glucose and insulin, as well as insulin resistance diagnostics (HOMA-IR) not only for T2DM but also for AD patients, as they are considered important prognostic tools for early AD detection and treatment [136]. As presented in this review, there is strong evidence indicating a close pathophysiological link between AD and T2DM. This close connection may lead to new common therapeutic perspectives, as various antidiabetic treatments are currently studied in several clinical studies for AD treatment. However, further research is needed in order to fully elucidate the shared molecular and pathophysiological mechanisms of AD and diabetes termed ‘Type-3-Diabetes’.

## Figures and Tables

**Table 1:** Sample of studies suggesting a link between type 2 diabetes mellitus and Alzheimer’s disease. (AGE: Advanced Glycosylation End; 4-HNE: 4-Hydroxynonenal; MDA: Malonaldehyde; 8-OHdG: 8-hydroxy-2’-deoxyguanosine; OSI: Oxidative Stress Index).

Study	Mechanism	Synopsis
Rivera et al. [20]	Low insulin and a decrease in choline acetyltransferase	Low insulin levels and sensitivity can lead to AD through a decrease in acetylcholine synthesis.
2. Janson et al. [21]	Amyloid deposition in islet and brain cells	Higher levels of islet amyloid in AD patients than in control subjects. No greater brain amyloid in DM patients compared with control subjects.
3. Miklossy et al. [19]	Amyloid beta and hyperphosphorylated tau	The presence of tau protein and β-amyloid in the pancreas after analysis of pancreatic tissue from 21 autopsy cases of patients with T2DM.
4. Kleinridders et al. [22]	Insulin resistance in the brain	Demonstrating a potential molecular link between central insulin resistance and behavioral disorders (in mice).
5. Maciejczyk et al. [23]	Oxidative stress	Significant increase in the oxidative damage markers (AGE, 4-HNE, MDA, 8-OHdG, and OSI) in the cerebral cortex of insulin-resistant mice.
6. Whitmer et al. [24]	Hypoglycemia	In older patients with T2DM, history of severe hypoglycemic episodes was associated with a greater risk of dementia.
7. Moran et al. [25]	T2DM and brain atrophy	T2DM is associated with reduced volume of the hippocampus.
8. An et al. [26]	Impaired glucose metabolism	Abnormalities in the metabolism of brain glucose may be intrinsic to the pathology of AD.
9. Sato and Morishita [27]	Mitochondrial function in the brain	T2DM impairs neurite outgrowth through JAK/STAT3 modulation of mitochondrial bioenergetics.
